# Interferon alpha inhibits antigen-specific production of proinflammatory cytokines and enhances antigen-specific transforming growth factor beta production in antigen-induced arthritis

**DOI:** 10.1186/ar4326

**Published:** 2013-10-03

**Authors:** Jaya Prakash Chalise, Sudeep Chenna Narendra, Bhesh Raj Paudyal, Mattias Magnusson

**Affiliations:** 1 Department of Clinical and Experimental Medicine, Linköping University, 581 83 Linköping, Sweden

## Abstract

**Introduction:**

Interferon alpha (IFN-α) has a complex role in autoimmunity, in that it may both enhance and prevent inflammation. We have previously shown that the presence of IFN-α at sensitization protects against subsequent antigen-triggered arthritis. To understand this tolerogenic mechanism, we performed a descriptive, hypothesis-generating study of cellular and humoral responses associated with IFN-α-mediated protection against arthritis.

**Methods:**

Arthritis was evaluated at day 28 in mice given a subcutaneous injection of methylated bovine serum albumin (mBSA), together with Freund adjuvant and 0 to 5,000 U IFN-α at days 1 and 7, followed by intraarticular injection of mBSA alone at day 21. The effect of IFN-α on mBSA-specific IgG1, IgG2a, IgG2b, IgA, and IgE was evaluated by enzyme-linked immunosorbent assay (ELISA). Cytokines in circulation and in *ex vivo* cultures on mBSA restimulation was evaluated with ELISA and Luminex, and the identity of cytokine-producing cells by fluorescence-activated cell sorting (FACS) analysis.

**Results:**

Administration of IFN-α protected mice from arthritis in a dose-dependent manner but had no effect on antigen-specific antibody levels. However, IFN-α did inhibit the initial increase of IL-6, IL-10, IL-12, and TNF, and the recall response induced by intraarticular mBSA challenge of IL-1β, IL-10, IL-12, TNF, IFN-γ, and IL-17 in serum. IFN-α decreased both macrophage and CD4^+^ T cell-derived IFN-γ production, whereas IL-17 was decreased only in CD4^+^ T cells. *Ex vivo*, in mBSA-restimulated spleen and lymph node cell cultures, the inhibitory effect of *in vivo* administration of IFN-α on proinflammatory cytokine production was clearly apparent, but had a time limit. An earlier macrophage-derived, and stronger activation of the antiinflammatory cytokine transforming growth factor beta (TGF-β) was observed in IFN-α-treated animals, combined with an increase in CD4^+^ T cells producing TGF-β when arthritis was triggered by mBSA (day 21). Presence of IFN-α at immunizations also prevented the reduction in TGF-β production, which was induced by the intraarticular mBSA injection triggering arthritis in control animals.

**Conclusions:**

Administration of IFN-α has a profound effect on the cellular response to mBSA plus adjuvant, but does not affect antigen-specific Ig production. By including IFN-α at immunizations, spleen and lymph node cells inhibit their repertoire of antigen-induced proinflammatory cytokines while enhancing antiinflammatory TGF-β production, first in macrophages, and later also in CD4^+^ T cells. On intraarticular antigen challenge, this antiinflammatory state is reenforced, manifested as inhibition of proinflammatory recall responses and preservation of TGF-β levels. This may explain why IFN-α protects against antigen-induced arthritis.

## Introduction

Type I interferon (IFN), mainly IFN-α and IFN-β, are important antiviral cytokines that also have complex roles in regulating inflammation. They may enhance immune responses contributing to effective viral clearance, but excessive activation of type I IFN production may lead to chronic inflammatory conditions, such as systemic lupus erythematosus (SLE) [1]. Conversely, type I IFN can dampen inflammatory conditions such as multiple sclerosis MS [2] and experimental models of colitis [3]. We earlier showed that injection of interferogenic dsRNA or IFN-α into a healthy mouse joint induces transient arthritis, which may explain why arthritis may follow viral infections [4]. In contrast, we also showed that if interferogenic dsRNA or IFN-α is coadministered at repeated immunizations with methylated bovine serum albumin, it totally prevents subsequent induction of antigen-induced arthritis [5].

Inhibition of IFN-α may be a future treatment modality against SLE (Sifalimumab) [6], and IFN-β is currently an important treatment against MS [7]. The factors determining whether type I IFN will act as a pro- or antiinflammatory are, however, not established. To develop safer therapeutics and to isolate the pro- or antiinflammatory properties of type I IFN (which could broaden the therapeutic applications of modulation of type I IFN signaling), it is important to understand the mechanisms behind its pro- and antiinflammatory effects.

The pro- and antiinflammatory effects comprise both innate and adaptive immunity. The ability of dsRNA to induce arthritis if deposited in the joint is critically dependent on type I IFN signaling [4] and does not require adaptive immunity [8]. In contrast, in antibody-induced arthritis, which also can occur in mice devoid of adaptive immunity [9], administration of IFN-α or dsRNA protects against arthritis development [10,11]. Thus, dependent on the location and mode of administration, the effect of IFN-α on innate immune mechanisms may be both pro- and antiinflammatory.

The proinflammatory effect of IFN-α in adaptive immunity is well illustrated by the enhancing effect of IFN-α on Th1-responses in SLE and models thereof [12]. By studying the effects of IFN-α released from plasmacytoid dendritic cells on Th-responses in SLE, Seventer and co-workers [13] proposed a pathogenic role for IFN-α in the triggering of the disease, whereas in established disease, and in chronic viral infections with continued type I IFN activation, IFN-α rather inhibits development of proinflammatory Th responses [13]. This is manifested by inhibited Th1 and Th17 development by IFN-α, which may be an important factor in limiting the tissue damage in SLE and chronic viral infections [13]. Likewise, the therapeutic effect of type I IFN in MS, and in experimental models of inflammatory bowel disease is thought to be mediated via an inhibitory effect of type I IFN on Th1 and Th17 development [14].

Antigen-induced arthritis (AIA) is an experimental model of RA that involves generation of antibodies and T cells with the capacity to transfer the disease [15]. Arthritis is induced by intraarticular injection of methylated bovine serum albumin (mBSA) in animals preimmunized with mBSA 2 and 3 weeks earlier. If IFN-α is administered along with the antigen at immunizations, animals fail to develop arthritis on intraarticular injection of mBSA [5]. The work of our laboratory is focused on determining the mechanism behind this antiinflammatory effect of IFN-α. Downmodulation of AIA may involve modulation of the anti-mBSA antibody repertoire from IgG2a and 2b toward IgG1 and IgA, which are less inflammatory [16]. Likewise, induction of mucosal IgA against CCP is associated with less-severe RA [17]. Also, in Lyme-induced arthritis, treatment with subtoxic doses of inorganic mercury clearly ameliorates arthritis, while increasing circulating levels of total IgE [18]. Type I IFN signaling may enhance antigen-specific immunoglobulin levels of most IgG subclasses [19] and IgA [20], whereas both enhancing [21] and dampening [22] effects of IFN-α on IgE levels have been observed.

A major contributor to joint inflammation in RA and AIA is proinflammatory cytokines. IL-1β, IL-6, IL-17, and TNF are found to be elevated during the development of arthritis [23], and inhibition of TNF and IL-6 represent successful treatments of RA [24,25]. Conversely, antiinflammatory cytokines (for example, IL-10 [26], TGF-β [27], and IL-13 [28]) may dampen arthritis.

To understand the mechanism of IFN-α-mediated tolerance induction, we performed a descriptive, hypothesis-generating study on how IFN-α affects the generation of humoral and cellular anti-mBSA responses during the induction of arthritis. We report here that IFN-α protects against arthritis in a dose-dependent manner and imprints leukocytes, first macrophages, and later also T-helper cells to release antiinflammatory TGF-β, while inhibiting proinflammatory cytokine release at encounter with antigen.

## Methods

### Mice

Female Sv129EV and A129 (mice deficient for the subunit 1 of the type I IFN receptor, IFNAR ko), B & K Universal (Aldbrough, UK) and Balb/c (B&K, Stockholm, Sweden) mice aged 8 to 12 weeks were kept under standard conditions of temperature and light, fed standard food and water *ad libitum* at the Animal Housing Unit of the Faculty of Health sciences, Linköping University. The experiment was approved by the Ethical Committee Board, Linköping University (Dnr 77–09). All experimental procedures were performed according to the Swedish Animal Welfare Act.

### Arthritis induction

AIA was induced as described by Van den Berg *et al.*[29] with some modifications. Briefly, mice were subcutaneously immunized at day 1 in the flank with 200 μg of methylated bovine serum albumin (mBSA) (Sigma-Aldrich, Munich, Germany) emulsified in incomplete Freund adjuvant (IFA) (Sigma, St. Louis, MO,USA), if not indicated otherwise, with 0, 100, 1,000, or 5,000 U of recombinant IFN-α (cat. no. 12100–1; PBL, Interferon Source, Piscataway, NJ, USA). The primary immunization was followed by a booster immunization in the tail with 100 μg of mBSA (prepared as on day 1 in IFA, if not indicated otherwise, with or without IFN-α) at day 7. At day 21, 30 μg of mBSA dissolved in 20 μl PBS was given intraarticularly in the left knee, and the same amount of PBS was injected in the right knee. On day 28, the mice were killed by cervical dislocation under anesthesia. Blood samples were collected at days 0, 14, 21, and 28 from the tail vein or by cardiac puncture. After centrifugation at 2,500 *g*, serum was separated and stored at −20°C until use.

### Histologic examination

The knee joints were removed from the killed mice at day 28 and fixed in 4% formaldehyde for 7 days, decalcified, dehydrated, and embedded in paraffin. Sagittal knee sections (4 μm) were stained with hematoxylin and eosin. A blinded observer (JP) scored arthritis from 0 to 3, as earlier described [5].

### Antibody analysis

Enzyme-linked immunosorbent assay (ELISA) plates (96-well, flat-bottomed, NUNC) were coated overnight at 4°C with mBSA (10 μg/ml) diluted in coating buffer (50 m*M* bicarbonate/carbonate buffer). The plate was blocked with 2% casein (Sigma) overnight. Serum samples were diluted in casein buffer 1:500 for determination of IgG1, IgG2a, and IgG2b, and 1:20 for IgA and IgE. Then 100 μl of diluted serum samples was added in triplicate and incubated for 2 hours. Then 100 μl of horseradish peroxidase (HRP)-conjugated secondary antibody goat anti-mouse IgG1, IgG2a, IgG2b, IgE (Bethyl Laboratories, Montgomery, AL, USA) or rat anti-mouse IgA (Southern Biotech, Birmingham, AL, USA) was added and incubated for 2 hours. The antibodies were diluted in casein buffer at the concentration as specified for ELISA by the manufacturer. Washing was performed 3 times in each step with phosphate-buffered saline (PBS) + 0.05% Tween-20. The plate was developed by adding 100 μl of tetra 3,3′,5,5′-tetramethylbenzidine (Sigma) solution, followed by incubation (dark) for 15 minutes. Finally, the reaction was stopped with 50 μl 1 *M* H_2_SO_4_, and the developed color was quantified at 450 nm.

### *Ex vivo* restimulation of splenocytes and lymph node cells

The spleens and a pool of lymph nodes draining the site of the first immunization (axillary) and the site of the second immunization and the knee joint (brachial, popliteal, and inguinal) were collected at days 0, 14, 21, and 28 of AIA. Spleens and lymph nodes were crushed and passed through a 70-μm nylon cell strainer. Red blood cells were lysed by using RBC-lysing solution (Sigma-Aldrich). The cells were resuspended in Iscove Modified Dulbecco Media (Sigma-Aldrich) supplemented with 10% heat-inactivated fetal bovine serum, 4 m*M* glutamine, 50 μ*M* β-mercaptoethanol, 100 U/ml penicillin, and 0.1 mg/ml streptomycin (Sigma-Aldrich). Then 100 μl of 2 × 10^6^ cells per ml of the spleen and pooled lymph node cells were cultured for 48 hours in triplicate, stimulated with 100 μl of mBSA solution (50 μg/ml). This concentration was based on previous titration of the proliferative response *ex vivo*[5].

### Cytokine analysis

The levels of IL-1β, IL-6, IL-17, IL-12, IFN-γ, TNF-α, IL-10, and IL-13 in serum collected at days 14, 21, and 28 and in supernatants from *ex vivo* antigen-stimulated splenocytes and lymph node cells collected after 48 hours of culture were determined with the Multiplex Luminex kit (Bio-Rad Laboratories, USA). The procedure was followed strictly according to the protocol provided by the company. The analytes were read with a Luminex-200 machine (Invitrogen), and the data analysis was done with MasterPlex 2010 (version 5.0.0.68). TGF-β1 levels in serum and in supernatant from splenocytes and lymph nodes culture were determined with ELISA (eBioscience, San Diego, CA, USA), according to manufacturer’s instructions.

### FACS analysis

Spleen and draining lymph nodes cells isolated at days 0, 14, 21, and 28 from mice during AIA were restimulated *ex vivo* with medium, mBSA, and anti-CD3 for 24 hours. Brefeldin A (5 μg/ml) and monensin (1 μg/ml; Biolegend, San Diego, CA, USA) were added 5 hours before harvest. Cells were resuspended (5 million cells/ml) in PBS containing 0.5% FBS, blocked with anti-mouse CD16/32 (Biolegend), and surface stained with anti-CD4-FITC (Milteyeni Biotech, Lund, Sweden), anti-F4/80-AF700 (AbD Serotec, Dusseldorf, Germany), and diluted according to the manufacturers’ recommendations. For intracellular staining, the cells were further fixed and permeabilized with intracellular staining buffer set (eBioscience), according to the manufacturer’s instructions, and stained with anti-IFN-γ-APC (Biolegend), anti-IL-17A-PE (Biolegend), and anti-TGF-β-BV (Biolegend), diluted according to the manufacturer’s recommendations. Between each of these steps, cells were washed 3 times in PBS containing 0.5% FBS. The cells were analyzed with FACS Gallios (Beckman Coulter, Inc., Brea, CA, USA), and data were analyzed with Kaluza Flow Analysis Software, Beckman Coulter (version 1.2). The percentages of IFN-γ, IL-17A, and TGF-β-positive cells among CD4- and F4/80-positive cells (based on 10,000 to 25,000 gated events) were determined by FMO gating, as earlier described [30].

### Statistical analysis

All the data were analyzed by using Graph Pad Prism, Version 5.01. For comparison of arthritis severity, antibody and cytokine levels of two groups with different treatments, the nonparametric Mann–Whitney *U* test was used. The effect of IFN-α on the number of cytokine-producing cells was evaluated by the Student *t* test comparing percentages of CD4^+^ and F4/80^+^ cells (based on 10,000 to 25,000 gated events) expressing IFN-γ, IL-17, and TGF-β. The Fisher Exact probability test was performed to compare the frequency of arthritis within two groups. Differences are judged significant when the *P* value is less than 0.05.

## Results

### Interferon-alpha protects against AIA in a dose-dependent manner

**Figure 1:**
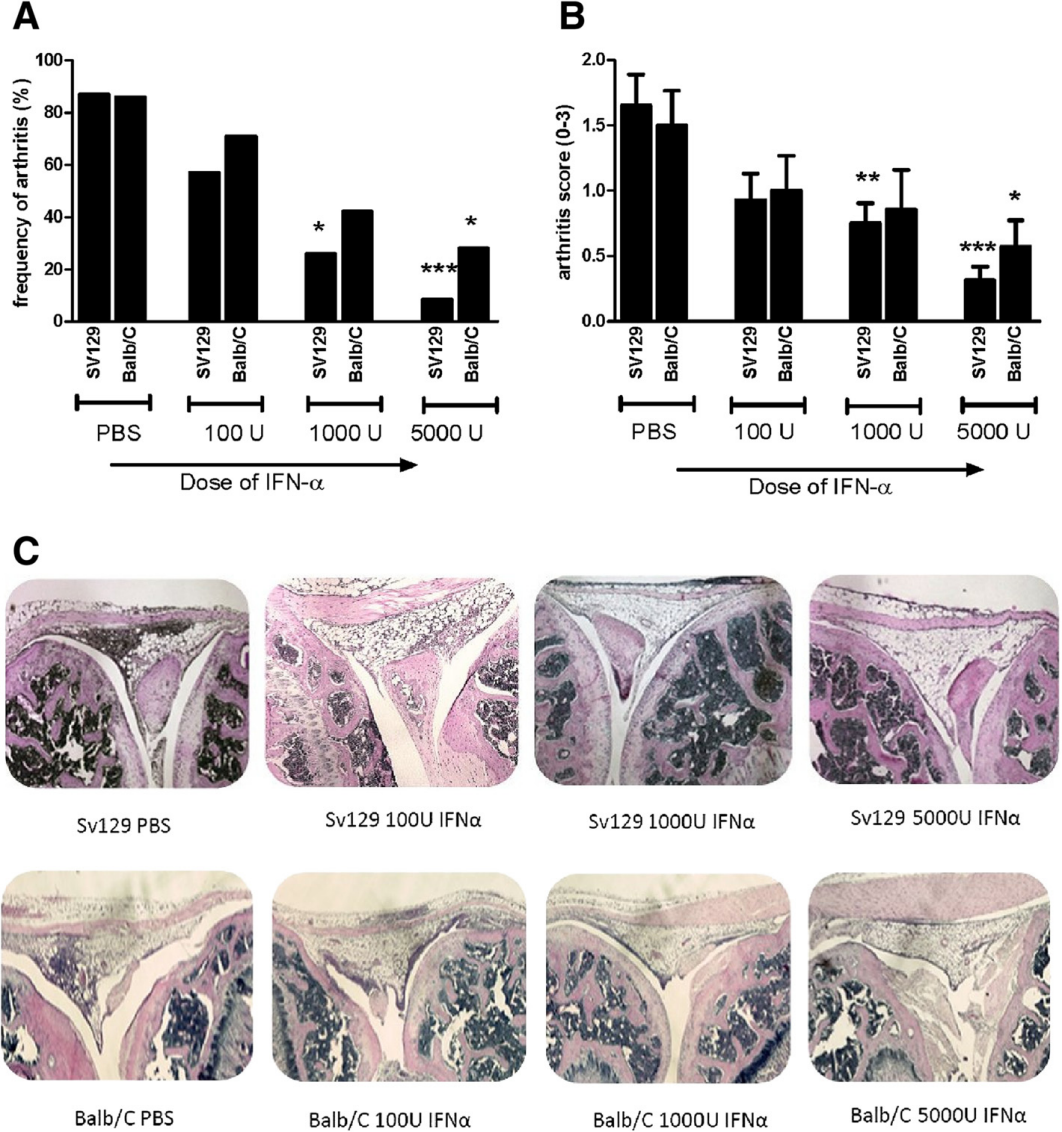
**Interferon alpha protects against AIA in a dose-dependent manner.** Female mice of strains SV129EV and Balb/c were immunized with mBSA at days 1 and 7 in Freund incomplete adjuvant mixed with 0, 100, 1,000, and 5,000 U IFN-α followed by intraarticular injection of same antigen in PBS at day 21. At day 28, mice were killed, histochemical sections of the knee joint were prepared, and arthritis was evaluated as described in "Methods". **(A)** Percentage of animals developing arthritis. **(B)** Arthritis severity (mean ± SEM). **(C)** Representative histochemical slides of the knee joints from each group. Severity of arthritis was evaluated by using the Mann–Whitney *U* test (*P < 0.05; **P < 0.01, compared with control (PBS) group; *n* = ≥6. The Fisher Exact test was performed to compare the arthritis frequency of interferon-treated groups and the corresponding control (PBS) group.

First we confirmed the ability of IFN-α to protect against mBSA-induced arthritis in the previously used strain, Sv129EV [5], and in the commonly used Balb/c strain. Histopathologic analysis of knee joints 1 week after intraarticular injection of mBSA confirmed that inclusion of 1,000 U of IFN-α at each of the two mBSA immunizations was clearly protective (Figure 1) and showed that the degree of arthritis protection was dependent on the dose of IFN-α. The frequency of arthritis was 90%, 60%, 15%, 7% for SV129 mice receiving 0 U, 100 U, 1,000 U, and 5,000 U of IFN-α at each immunization, respectively. In Balb/c mice, the arthritis incidence was 87%, 71%, 42%, and 28% in mice receiving 0 U, 100 U, 1,000 U, and 5,000 U of IFN-α, respectively (Figure 1). Thus, the higher dose (5,000 U IFN-α) was required to protect Balb/c mice fully from AIA; 100 units of IFN-α was found to be insufficient to protect more than 40% of mice in any strain of mice.

### IFN-α has limited effect on mBSA-specific humoral response

**Figure 2:**
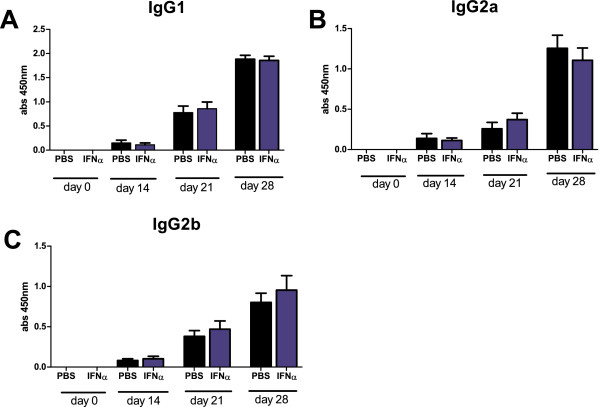
**No effect of IFN-α on antigen-specific IgG subtypes levels induced by mBSA plus IFA.** Mice were immunized twice with mBSA plus IFA with or without IFN-α in 1-week intervals followed by intraarticular injection of mBSA in PBS 21 days later. Serum was collected at days 0, 14, 21, and 28 and analyzed for mBSA-specific antibodies with ELISA by using detection antibodies specific for **(A)** IgG1, **(B)** IgG2a, and **(C)** IgG2b. Black bars represent control group without IFN-α treatment, and blue bars represent the IFN-α-treated group. Data are expressed as the mean absorbance (450 nm) ± SEM; *n* ≥ 12 (pooled data of two independent experiments).

**Figure 3:**
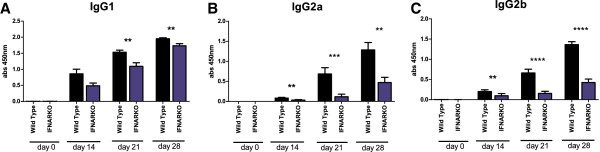
**Endogenous type I IFN signaling regulates the production of antigen-specific IgG subtypes induced by mBSA plus IFA.** Wild-type mice and mice unable to express functional type I receptor (IFNARKO) were immunized with mBSA plus IFA twice in a 1-week interval followed by intraarticular injection of mBSA in PBS 21 days later. Serum was collected at days 0, 14, 21, and 28 and analyzed for mBSA-specific antibodies with ELISA by using detection antibodies specific for **(A)** IgG1, **(B)** IgG2a, and **(C)** IgG2b. Data are expressed as the mean absorbance (450 nm) ± SEM; *n* ≥ 10. (*P < 0.05; **P < 0.01; ***P < 0.001; and ****P < 0.0001).

Increasing levels of antigen-specific IgG1, IgG2a, and IgG2b were detected during the course of AIA, but no significant difference was found between control mice and mice treated with 1,000 U IFN-α (Figure 2A,B,C) or 5,000 U IFN-α (data not shown). This was also apparent in the absence of adjuvant, in which immunization with mBSA alone resulted in low but significant antigen-specific production of total IgG and IgG1, which was not increased in the presence of 1,000 U IFN-α (see Additional file 1: Figure S1). In contrast, endogenous type I IFN signaling enhanced the IgG response because WT mice produced significantly higher levels of all analyzed IgG subtypes than did IFNAR ko mice, although IgG1 levels were clearly enhanced in both strains at day 28 (Figure 3).

**Figure 4:**
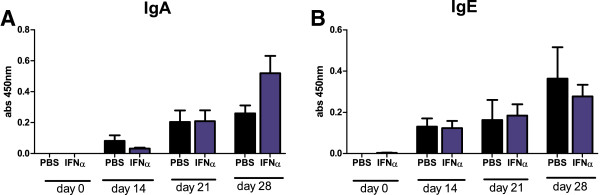
**No effect of IFN-α on antigen-specific IgA or IgE levels induced by mBSA plus IFA.** Mice were immunized twice with mBSA plus IFA with or without 1,000 U IFN-α in 1-week intervals followed by intraarticular injection of mBSA in PBS 21 days later. Serum was collected at days 0, 14, 21, and 28 and analyzed for mBSA-specific antibodies with ELISA by using detection antibodies specific for **(A)** IgA and **(B)** IgE. Black bars represent the control group without IFN-α treatment, and blue bars represent the IFN-α-treated group. Data are expressed as the mean absorbance (450 nm) ± SEM; *n* ≥ 12 (pooled data of two independent experiments).

Low, compared with IgG, but significantly higher than day zero levels of antigen-specific IgA were detected irrespective of IFN-α treatment in all mice in the prechallenge phase (Figure 4A). Thereafter, the levels steadily increased until arthritis manifestation, but no significant difference was observed between IFN-α-treated animals and controls. Significant increases of mBSA-specific IgE levels were detected at days 14, 21, and 28, but again, no significant difference was found between mice treated with IFN-α and nontreated mice (Figure 4B).

### Interferon-alpha inhibits the initial increase and the antigen-induced recall response of proinflammatory cytokines in serum during AIA

**Figure 5:**
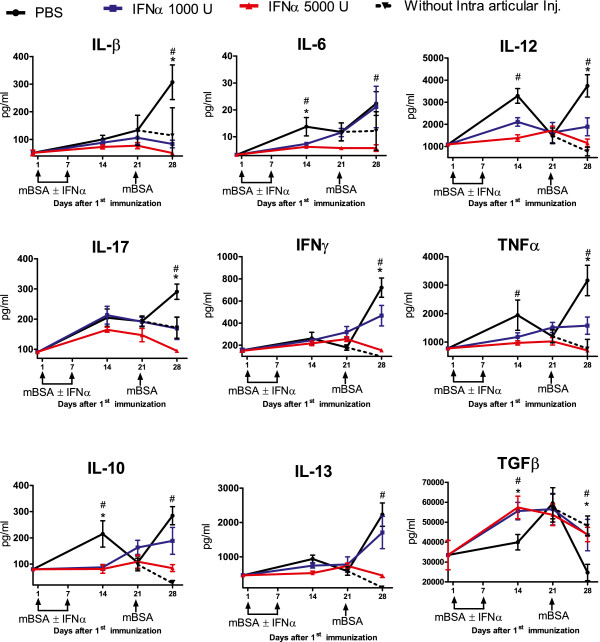
**IFN-α downmodulates initial activation and subsequent antigen-induced reactivation of proinflammatory cytokines but enhances the initial activation and prevents subsequent antigen-induced inhibition of TGF-β reactivation.** Mice were immunized twice with mBSA plus IFA with or without IFN-α at 1-week intervals followed by intraarticular injection of mBSA in PBS 21 days later. Levels of IL-1β, IL-6, IL-12, IL-17, TNF, IFN-γ, IL-10, and IL-13 were determined by Luminex, and TGF-β, by ELISA in serum collected at days 0, 14, 21, and 28 during the course of AIA. Data are expressed as mean pg/ml ± SEM (*n* ≥ 6). The black line, blue line, and red line represent groups receiving 0 U, 1,000 U, and 5,000 U of IFN-α, respectively, at the time of mBSA immunizations. Mann–Whitney *U* test was performed to compare cytokine levels between two groups. Animals not receiving intraarticular injection of mBSA (the dotted line) produced significantly (*P* < 0.05) less of all analyzed cytokines at day 28 compared with the control group. *P < 0.05 between PBS (black) and IFN-α 1,000 U (blue). #*P* < 0.05 between PBS (black) and IFN-α 5,000 U (red). The experiment was performed twice with similar results.

Two weeks after mBSA sensitization, the cytokines IL-1β, IL-6, IL-17, IL-12, IFN-γ, and TNF were increased in control mice (Figure 5, day 14). Thereafter, the level of cytokines in the control group diminished in general, which was manifested in significantly lower levels of IL-17, IL-12, and TNF at day 21, whereas IL-1β and IFN-γ remained unchanged between days 14 and 21 (Figure 5). One week after intraarticular injection of mBSA in preimmunized mice, the levels of IL-β, IL-6, IL-12, IL-17, IFN-γ, and TNF were increased compared with day 21 (Figure 5). The cytokines levels decreased or remained constant at day 28 in preimmunized mice that did not receive intraarticular injection (compare black and dotted lines in Figure 5).

The described cytokine profile was altered by IFN-α in a dose-dependent manner both in the prechallenge phase and at the time of arthritis manifestation. First, in mice receiving 5,000 U IFN-α at each immunization, the production of IL-6, IL-12, and TNF was significantly lower than in controls 2 weeks after the first immunization (Figure 5, day 14), and the 1,000 U dose of IFN-α resulted in significantly lower levels of IL-12 and a trend of lower IL-6 and TNF (Figure 5, day 14). Second, immunization in the presence of IFN-α clearly hampered or totally prevented the reactivation of cytokine release induced by intraarticular injection of mBSA. Presence of 1,000 U at each immunization totally prevented antigen-induced reactivation of IL-1β, IL-12, and TNF, and significantly reduced the antigen-induced levels of IL-17 and IFN-γ (Figure 5, day 28). Presence of 5,000 U IFN-α prevented antigen-induced recall responses of all proinflammatory cytokines (Figure 5, day 28).

### Interferon-α increases serum levels of TGF-β and prevents antigen-induced inhibition of TGF-β production

IFN-α effectively turned down the mBSA-induced proinflammatory cytokine response (Figure 5). Presence of IFN-α affected the serum levels of antiinflammatory IL-10 in the same way as it affected the majority of proinflammatory cytokines (that is, inhibited the initial activation (Figure 5, day 14) and the mBSA-induced recall response (Figure 5, day 28). Serum levels of IL-13 only reached above baseline on intraarticular injection of mBSA, and this activation was inhibited only by the higher dose of IFN-α. In apparent contrast to the effect on proinflammatory cytokines and IL-10, the presence of IFN-α resulted in significantly higher serum levels of TGF-β 2 weeks after the first immunization (Figure 5). One week later, TGF-β in the control group (that is, without IFN-α) was level with that in the IFN-α-treated group, but declined rapidly on intraarticular antigenic rechallenge (compare black and dotted lines in Figure 5). This rapid mBSA-induced decline in serum levels of TGF-β was effectively prevented by immunization in the presence of IFN-α. As depicted in Figure 5, IFN-α-treated groups had significantly higher serum levels of TGF-β at day 28 than did control mice.

### *In vivo* administration of interferon-α inhibits antigen-induced release of proinflammatory cytokines but enhances TGF-β production *ex vivo*

**Figure 6:**
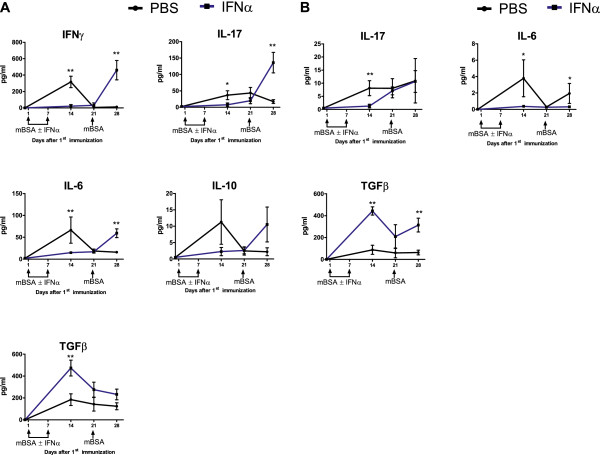
**IFN-α modulates antigen-induced cytokine production from splenocytes and lymph node cells *ex vivo*.** Spleens and draining lymph nodes were collected from mice at days 0, 14, 21, and 28 during the course of AIA. Single-cell suspensions were prepared and were restimulated with 50 μg/ml of mBSA for 48 hours. The supernatant of the culture was analyzed for different cytokines with Luminex (IL-1β, IL-6, IL-10, IL-12, IL-13, IL-17, IFN-γ, and TNF) and ELISA (TGF-β). The data are expressed as mean pg/ml ± SEM (*n* ≥ 6) in **(A)** splenocytes and **(B)** lymph node cells (*n* ≥ 6). From each value, the background cytokine levels found in culture media (including fetal bovine serum) were subtracted. The black line and the blue line represent groups receiving 0 U and 1,000 U of IFN-α, respectively, at the time of mBSA immunizations. Mann–Whitney *U* test was performed to compare cytokine levels between the two groups (*P < 0.05; **P < 0.01).

The serum cytokine profile observed during the development of mBSA-immunity and mBSA-triggered arthritis was heavily altered by immunization in the presence of IFN-α (Figure 5). Also, spleen and lymph node cells restimulated with mBSA *ex vivo* 2 weeks after the first immunization produced significant amounts of IL-6, IL-17, and TGF-β, whereas IFN-γ and IL-10 were detectable only in spleen cell cultures (Figure 6A,B). IL-1β, IL-12, IL-13, and TNF were not detectable (data not shown). At this time, reactivation *ex vivo* of IL-6, IL-10, IL-17, and IFN-γ, but not TGF-β, was totally inhibited in cells from mice receiving IFN-α at immunizations. Conversely, at this time, TGF-β production *ex vivo* was significantly enhanced in both lymph and spleen cells isolated from IFN-α-treated mice (Figure 6A,B, day 14, blue line).

### The inhibitory effect on *ex vivo* cytokine production of *in vivo* administration of IFN has a time limit

Spleen cells isolated from IFN-α-treated mice, whose serum levels of proinflammatory cytokines at day 28 were generally lower than those in control mice (Figure 5), readily produced IL-6, IL-17, and IFN-γ on antigenic restimulation at day 28 (Figure 6A). This waived inhibition was also observed in lymph node cell cultures for IL-17, but not for IL-6 (Figure 6B). Thus, the inhibitory effect on *ex vivo* splenocyte cytokine production observed 7 to 14 days after the last injection of IFN-α (Figure 6A) was not apparent 1 week later. Still, spleen and lymph node cells from IFN-α-treated mice produced higher levels than did cells from control mice of antiinflammatory TGF-β in response to mBSA-restimulation at this time point (significant for lymph node cells and a trend for splenocytes).

At day 28, when the serum cytokines (except TGF-β) levels peaked in the control group (see Figure 5), spleen cells were unexpectedly not responsive to mBSA-restimulation *ex vivo*, except for a weak ability to produce TGF-β (Figure 6A). This loss of activity was not observed in lymph node cells, which still produced IL-17 and IL-6 on restimulation *ex vivo* (Figure 6B).

### IFN-α regulates TGF-β and IFN-γ production in macrophages, and TGF-β, IL-17, and IFN-γ production in T cells

**Figure 7:**
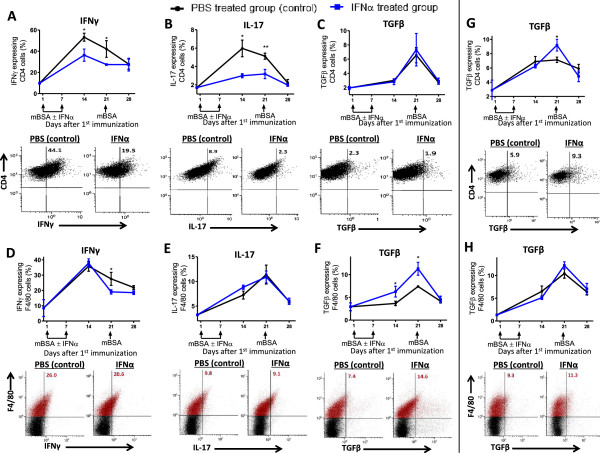
**IFN-α regulates TGF-β and IFN-γ production in macrophages and TGF-β, IL-17, and IFN-γ production in T cells.** Draining lymph nodes and spleen cells were collected from mice at days 0, 14, 21, and 28 during the course of AIA and restimulated with mBSA (50 μg/ml) for 24 hours. Brefeldin A (5 μg/ml) and monensin (1 μg/ml) were added 5 hours before FACS analysis. **(A-F)** Percentage of IFN-γ, IL-17A, and TGF-β expressing lymph node cells of CD4^+^ cells **(A-C)** and F4/80^+^ cells **(D, F)**. **(G**, **H)** percentage TGF-β-expressing spleen cells of CD4^+^ cells **(G)** and F4/80^+^ cells **(H)** during the course of AIA. The data shown are mean values ± SEM of pooled data from two independent experiments (*n* ≥ 5) with similar results. *P < 0.05; **P < 0.01. Blue line, mice immunized in the presence of IFN-α; black line, control. Below each graph is a representative dot plot of the same cells from mice with or without IFN-α treatment during AIA at day 14 **(A-C)** and day 21 **(D-H)**. For CD4^+^ cells, only gated cells are depicted. F4/80^+^ gated cells are depicted in red, and the figure in the upper right quadrant represents the percentage of cytokine-producing cells among F4/80 (red) cells.

Intracellular staining for TGF-β, IL-17, and IFN-γ in CD4^+^ and F4/80^+^ gated lymph node cells restimulated *ex vivo* with mBSA for 24 hours revealed that mBSA-immunization in the presence of IFN-α inhibited IFN-γ in both T cells (Figure 7A, days 14 and 21) and macrophages (Figure 7D, day 21), whereas IL-17 production was inhibited in CD4^+^ T cells (days 14 and 21, Figure 7B) but not in F4/80^+^ macrophages (Figure 7E). In spleen cells isolated from IFN-α-treated mice and restimulated with mBSA for 24 hours, a minor decrease in macrophages producing IFN-γ (Additional file 2: Figure S3D, day 14) and CD4^+^ T cells producing IL-17 was observed (Additional file 2: Figure S3B, day 14). IFN-α had no inhibiting effect on the number of IFN-γ- and IL-17-producing cells when lymph node and spleen cells were unstimulated (mock) or generally stimulated by anti-CD3 (see Additional file 3: Figure S2, and Additional file 2: Figure S3).

Presence of IFN-α at immunizations clearly augmented the number of TGF-β-producing F4/80^+^ macrophages, as depicted on days 14 and 21 of AIA for lymph node cells (Figure 7F) but had no effect on TGF-β production from CD4^+^ T cells in lymph node cells (Figure 7C). This enhancing effect in macrophages was not present in spleen cells, in which instead, an increase of TGF-β-producing CD4^+^ T cells was observed on day 21 (Figure 7G). The enhancing effect of IFN-α on TGF-β production in macrophages was also clearly apparent in mock-stimulated lymph node cells isolated from mice immunized in the presence of IFN-α. (Additional file 3: Figure S2F).

## Discussion

We recently showed that viral RNA, or IFN-α by itself, by activating type I IFN signaling, protects against antigen-induced arthritis [5]. The protection is dose dependent, and Balb/c mice required a higher dose IFN-α than did Sv129 mice to be protected (Figure 1). This may also reflect dose dependency, but in terms of the strength of type I IFN signaling, which affects AIA because IFNAR ko mice develop a much more severe arthritis than do WT mice [5]. Type I IFN production is critically dependent on positive feedback via the type I IFN receptor, and Sv129 mice have a strong type I IFN signaling, in that they can produce more IFN-α compared with other strains, including Balb/c [31]. The stronger endogenous type I IFN signaling therefore likely explains why Sv129 mice require less IFN-α than do Balb/c mice for protection against AIA.

The fact that IFN-α, while preventing development of mBSA-induced arthritis, had no effect on anti-mBSA antibody levels, questions (but does not formally exclude) the importance of humoral immunity in the pathogenesis of mBSA-induced arthritis. Early work by Brackertz [15] showed that serum from immunized mice could transfer arthritis susceptibility, but the disease was very mild in comparison with that observed on T-cell transfer or in immunized mice. It was later observed that mice deficient in mature B cells, and thus unable to secrete antibodies, are also susceptible to mBSA-induced arthritis [32,33]. Thus, mBSA antibodies contribute only a little, seemingly dispensable part of the pathogenesis of mBSA-induced arthritis.

Le Bon *et al*. [19] showed that administration of 10^5^ U IFN-α/β at immunization with chicken γ-globulin enhances the antibody response. Although the antigens and use of adjuvant differ, doses up to 5,000 U IFN-α seem not to have this effect because the levels of mBSA-specific total IgG, IgG1, IgG2a, and IgG2b did not increase by immunization in the presence of IFN-α. No enhancing effect of IFN-α was masked by the potentiating effect of IFA because IFN-α did not increase anti-mBSA immunoglobulins when mBSA was administered without IFA (Additional file 1: Figure S1). However, endogenous type I IFN signaling does regulate the establishment of humoral immunity activated by mBSA+IFA, as the levels of IgG1, IgG2a, and IgG2b were lower in IFNAR ko mice as compared with WT (Figure 3). Still, at arthritis manifestation (day 28), IFNAR ko mice produced significant levels of all analyzed antibodies, indicating that other signaling pathways clearly play an important role in establishing the full anti-mBSA IgG response.

A pathogenic factor in the development of arthritis is inflammation driven by proinflammatory cytokines, especially TNF, IL-1β, IL-6, and IL-17 [34]. Before the profound effect on cytokine production evoked by administration of IFN-α is detailed and related to arthritis protection, the general pattern of cytokines induced by immunization with mBSA plus IFA is shortly recapitulated.

The serum cytokine pattern induced by mBSA+IFA was characterized by a peak of proinflammatory TNF, IL-6, IL-12, IL-17, antiinflammatory IL-10 and IL-13, and immunomodulatory IFN-γ 2 weeks after the first immunization, followed by a significant decrease of the majority of these cytokines (IL-12, TNF, and IL-10, and a trend for IL-17, IL-13, and IFN-γ). A second peak of serum cytokine levels, concomitant with arthritis manifestation, was induced by intraarticular injection of mBSA (Figure 5). In contrast, TGF-β did not follow this pattern but was instead characterized by a delayed response that peaked 3 weeks after the first immunization and strikingly, rapidly declined below baseline levels on intraarticular injection of mBSA (that is, when arthritis was established (Figure 5)).

Interestingly, immunization in the presence of IFN-α totally reversed this cytokine pattern. Interferon-α inhibited both the initial activation and the second peak induced by mBSA of the majority of proinflammatory cytokines (Figure 5). In contrast, it enhanced and advanced the initial activation, and importantly, prevented antigen-induced downmodulation of antiinflammatory/immunomodulatory TGF-β (Figure 5). Immunization in the presence of IFN-α also reversed the cytokine pattern observed in *ex vivo* stimulated leukocyte cultures, in that it inhibited the early response of the majority of proinflammatory cytokines but enhanced that of TGF-β (Figure 6A,B).

At arthritis manifestation, when the serum levels of proinflammatory cytokines peaked in the arthritic control group (Figure 5), the numbers of IFN-γ- and IL-17-containing CD4^+^ and F4/80 cells were generally lower than at earlier times (Figure 7 and Additional file 3: Figure S2, and Additional file 2: Figure S3), and mBSA-restimulated spleen cells from these mice were totally unresponsive in terms of releasing proinflammatory cytokines (Figure 6A, day 28). The *in vivo* reactivation by mBSA challenge day 21, resulting in the second peak of proinflammatory cytokines, may thus have rendered them resistant to further mBSA stimulation. In contrast, production of TGF-β, which was clearly inhibited *in vivo* at arthritis manifestation in the control group (Figure 5), was not inhibited in spleen-cell cultures stimulated *ex vivo* with mBSA in the same way as proinflammatory cytokines (Figure 6A).

Similarly, whereas day 28 saw low levels of proinflammatory cytokines in mice receiving IFN-α (Figure 5), spleen cells isolated the same day were responsive to mBSA-restimulation *ex vivo* (Figure 6A). At earlier times, spleen cells restimulated *ex vivo* are still held back, despite lack of IFN-α in *ex vivo* cultures (Figure 6A). Thus, 3 weeks after the last injection of mBSA in IFA plus IFN-α, the inhibitory effect on proinflammatory cytokines is still present *in vivo*, but if spleen cells are restimulated *ex vivo*, the inhibition is waived. This shows that the effect of IFN-α may have a time limit and be reversible, at least *ex vivo*. It remains to be determined whether animals immunized in the presence of IFN-α are resistant to mBSA-induced increase of proinflammatory cytokines *in vivo* and arthritis at later time points.

Presence of IFN-α at immunizations thus inhibited production of the Th1-promoting cytokines IL-12 and TNF in the early phase (Figure 5) and IFN-γ, including macrophages producing IFN-γ at later times (Figures 5 and 7D), which is in line with the inhibitory effect of type I IFNs on Th1 immunity (IL-12, TNF, IL-1β, and IFN-γ signaling) in monocytes [35]. The early inhibition of Th1-promoting cytokines, especially IL-12 (Figure 5), may be important for the lower number of T-helper cells (CD4^+^) producing the Th1 cytokine IFN-γ (Figure 7A) in mice receiving IFN-α at immunizations. On mBSA challenge day 21, this low number of Th1 cells (Figure 7A) may result in the inhibited mBSA recall response of IFN-γ, IL-12, and TNF observed at day 28 in mice immunized in the presence of IFN-α (Figure 5).

IFN-α may drive Treg development [36,37], which has been demonstrated by administration of interferogenic CpG-DNA that supported the development of CD4^+^CD25^+^ T cells in an IFNAR-dependent manner [38]. An important mediator for development of both Tregs [39] and their suppressive capacity is the antiinflammatory cytokine TGF-β. Interestingly, TGF-β, in contrast to all other analyzed cytokines, was significantly increased in mice treated with IFN-α. The initial source of TGF-β that could shape an antiinflammatory state that prevents arthritis development is, however, not likely Tregs. Albeit not necessarily limited to that, an early source of TGF-β in mice treated with IFN-α was instead macrophages (Figure 7F and Additional file 3: Figure S2F), and not until day 21 was an increase in CD4^+^ T cells producing TGF-β observed in IFN-α-treated cells, as compared with control animals (Figure 7G). The ability of both TGF-β and IFN-α to induce Tregs is critically dependent on the cytokine microenvironment. At excessive inflammation (that is, in the presence of high levels of IL-6), TGF-β inhibits Treg development while promoting the generation of Th17 cells [40]. Likewise, in the presence of IL-6, CpG-DNA-activated pDC may contribute to Th17 development via both IFN-α and TGF-β [41]. Importantly, although TGF-β levels were significantly higher, the levels of IL-6 at day 14 in IFN-α-treated animals were significantly lower than those in the control group (Figure 5). Thus, by initially (day 14) activating TGF-β (Figure 5) in macrophages (Figure 7F), while keeping IL-6 at bay (Figure 5), type I IFN may favor TGF-β-dependent tolerance by regulatory T cells while preventing activation of TGF-β^+^ IL-6=induced Th17 differentiation. Indeed, at day 14 when the number of IL-17-producing CD4^+^ T cells in lymph nodes from control animals was tripled as compared with baseline, no significant increase was yet observed in IFN-α-treated mice (Figure 7B). Macrophages producing IL-17 during AIA (Figure 7E and Additional file 2: Figure S3E) were not inhibited by IFN-α, and it is therefore likely that the inhibited IL-17 recall response *in vivo* in IFN-α-treated animals is due mainly to the lower number of IL-17-producing CD4^+^ T cells (day 21, Figure 7B).

Once tolerance is established, antigen-restimulation may reactivate TGF-β production, both *in vivo*[42] and *ex vivo*[43,44]. In contrast, if inflammation is predominant, antigen restimulation may inhibit TGF-β production, producing effective immunity [45]. Likewise, in arthritic control animals, we observed a rapid decline in TGF-β levels in serum on intraarticular rechallenge with antigen, confirming a predominant inflammatory state. In contrast, in mice receiving IFN-α at immunizations, the decline in TGF-β at antigen rechallenge was prevented, and the TGF-β levels followed those of mBSA-immunized mice not receiving intraarticular injection of mBSA (Figure 5). The tolerance was also reflected in higher levels of TGF-β released from mBSA-stimulated lymph node and spleen cells from IFN-α-treated animals (Figure 6A,B). Thus, one effect of immunization with mBSA+IFA in the presence of IFN-α may be development of mBSA-specific tolerogenic cells, which, on intraarticular injection, are reactivated to produce TGF-β (Figure 6B), which prevents activation of mBSA-induced arthritis. The increase in CD4^+^ T cells producing TGF-β among spleen cells from IFN-α-treated mice at day 21 (Figure 3G) may represent such cells that, together with TGF-β-producing macrophages (Figure 7H), would give rise to the higher TGF-β levels observed in serum (Figure 5) and lymph-node cultures (Figure 6B) from IFN-α-treated mice at the day of arthritis manifestation. Possibly, continuous release of TGF-β may exhaust these cells, resulting in a rapid decline when assessing the number *in vitro* at day 28 (Figure 7). Further supporting a role of TGF-β in tolerance-induced protection against arthritis is that blocking of TGF-β compromises oral tolerance to collagen-induced arthritis [46].

## Conclusion

In this work, we showed that prevention of mBSA-induced arthritis by IFN-α is independent of modified antigen-specific antibody production but involves regulation of the mBSA-specific cellular response. The tolerogenic state induced by IFN-α is likely induced by a combination of inhibition of proinflammatory cytokines, especially early IL-6 production, with early enhancement of TGF-β-producing macrophages, which together result in fewer IFN-γ- and IL-17-producing CD4^+^ T cells and development of TGF-β-producing CD4^+^ T cells.

## Abbreviations

AIA: Antigen-induced arthritis; dSRNA: Double-stranded RNA; IFA: Incomplete freund adjuvant; IFN: Interferon; IL: Interleukin; mBSA: Methylated bovine serum albumin; RA: Rheumatoid arthritis; TGF: Transforming growth factor; TNF: Tumor necrosis factor.

## Competing interests

The authors declare that they have no competing interests.

## Authors’ contributions

JP performed laboratory and animal experimentation, contributed to the design of the study, and wrote the draft of the manuscript. SN performed laboratory and animal experimentation and contributed to the design of the study. BR performed laboratory work. MM designed the study and finalized the manuscript, together with JP. All authors read and approved the final manuscript.

## Supplementary Material

Additional file 1: Figure S1 Effect of IFN-α administration on antigenic specific total IgG and IgG subtypes during AIA in absence of adjuvant. Mice were injected twice with mBSA with or without 1,000 U IFN-α in 1-week intervals followed by intraarticular injection of mBSA in PBS 21 days later. Serum was collected at days 0, 14, 21, and 28 and analyzed for mBSA-specific antibodies with ELISA by using detection antibodies specific for total IgG, IgG1, IgG2a, and IgG2b. Black bars represent control group without IFN-α treatment, and blue bars represent IFN-α-treated group. Data are expressed as the mean absorbance (450 nm) ± SEM, *n* ≥ 6. Click here for file

Additional file 2: Figure S3 Effect of IFN-α on intracellular cytokine expression in mock, mBSA and anti-CD3-restimulated T cells and mock and mBSA-stimulated macrophages from splenocytes during AIA. Spleens were collected from mice at days 0, 14, 21, and 28 during the course of AIA and restimulated with complete media (mock) or mBSA (50 μg/ml) or anti-CD3 (1 μg/ml) for 24 hours. Brefeldin A (5 μg/ml) and monensin (1 μg/ml) were added 5 hours before FACS analysis. **(A-F)** show percentage IFN-γ-, IL-17A-, and TGF-β-expressing spleen cells of gated CD4^+^**(A-C)** and F4/80^+^ cells **(D-F)**. The data shown are mean values ± SEM of pooled data from two independent experiments (*n* ≥ 5) with similar results (**P < 0.01). Blue line, mice immunized in the presence of IFN-α; black line, control. Click here for file

Additional file 3: Figure S2 Effect of IFN-α on intracellular cytokine expression in mock-stimulated macrophages and mock- and anti-CD3-stimulated T cells from lymph nodes during AIA. Draining lymph node cells were collected from mice at days 0, 14, 21, and 28 during the course of AIA and restimulated with complete media (mock) or anti-CD3 (1 μg/ml) for 24 hours. Brefeldin A (5 μg/ml) and monensin (1 μg/ml) were added 5 hours before FACS analysis. **(A-F)** Percentage IFN-γ-, IL-17A-, and TGF-β-expressing lymph node cells of gated CD4^+^ (A-C) and F4/80^+^ cells (D-F). The data shown are mean values ± SEM of pooled data from two independent experiments (*n* ≥ 5) with similar results (*P < 0.05). Blue line, mice immunized in the presence of IFN-α; black line, control. Click here for file
