# Supplementary figures and images for: Interferon alpha inhibits antigen-specific production of proinflammatory cytokines and enhances antigen-specific transforming growth factor beta production in antigen-induced arthritis
Source: Arthritis Res Ther. 2013 Oct 3;15(5):R143. doi: 10.1186/ar4326 (PMC3978460; doi:10.1186/ar4326)

Figure S1

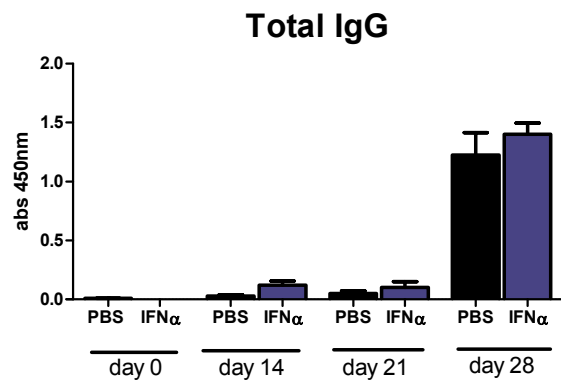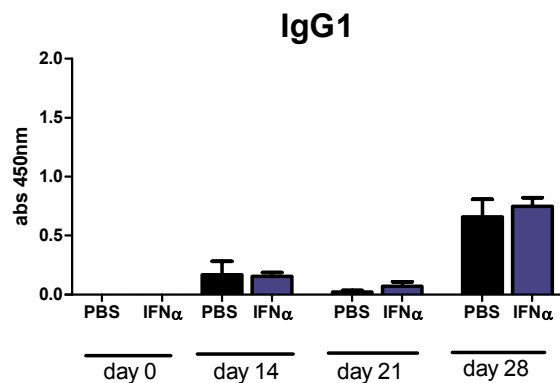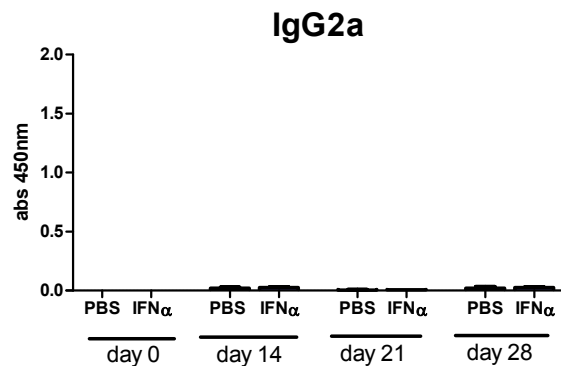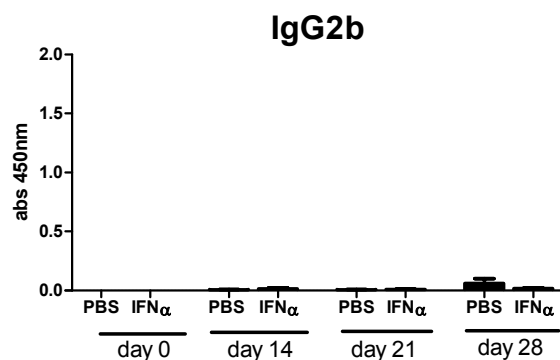

Supplement: Additional file 1: Figure S1 — Effect of IFN-α administration on antigenic specific total IgG and IgG subtypes during AIA in absence of adjuvant. Mice were injected twice with mBSA with or without 1,000 U IFN-α in 1-week intervals followed by intraarticular injection of mBSA in PBS 21 days later. Serum was collected at days 0, 14, 21, and 28 and analyzed for mBSA-specific antibodies with ELISA by using detection antibodies specific for total IgG, IgG1, IgG2a, and IgG2b. Black bars represent control group without IFN-α treatment, and blue bars represent IFN-α-treated group. Data are expressed as the mean absorbance (450 nm) ± SEM, n ≥ 6. [file ar4326-S1.pdf]

Figure S3

● PBS treated group (control) ■ IFN $\alpha$  treated group

**A**

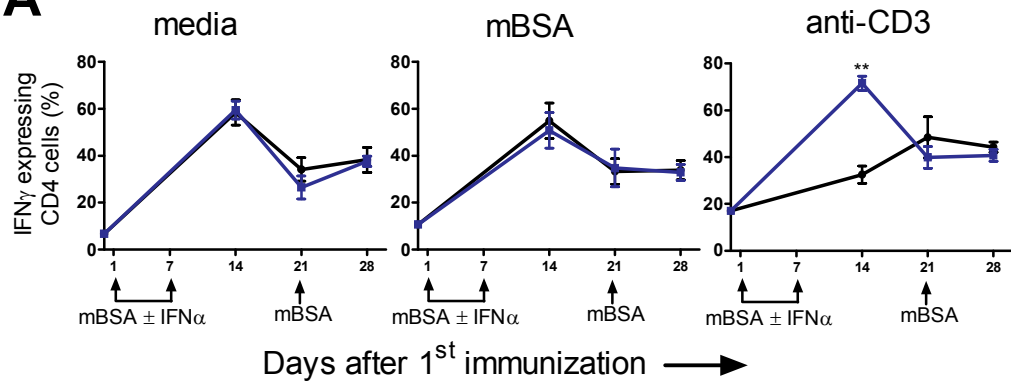

**D**

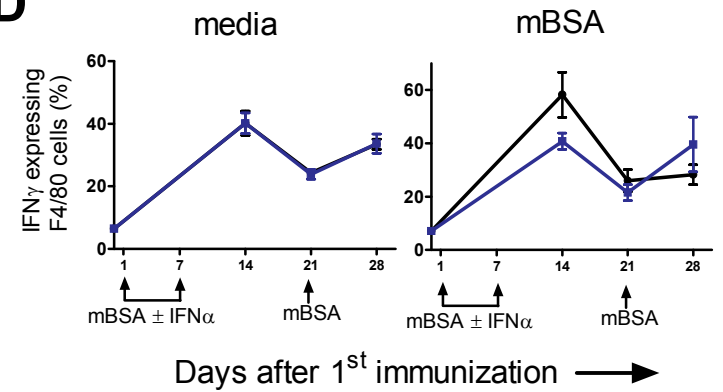

**B**

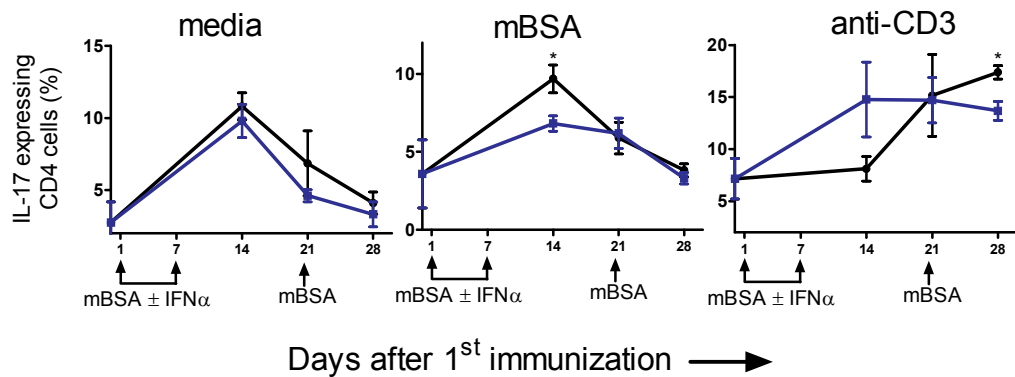

**E**

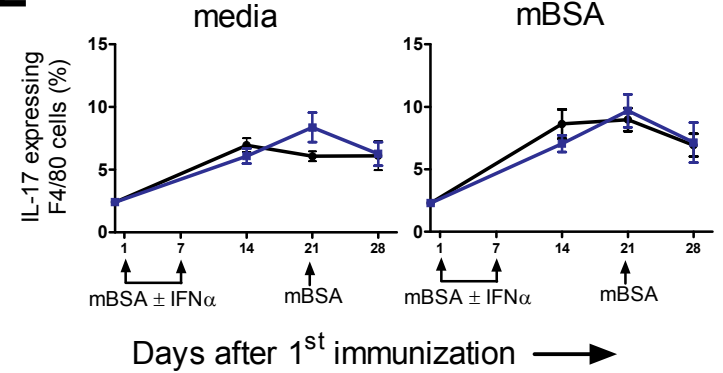

**C**

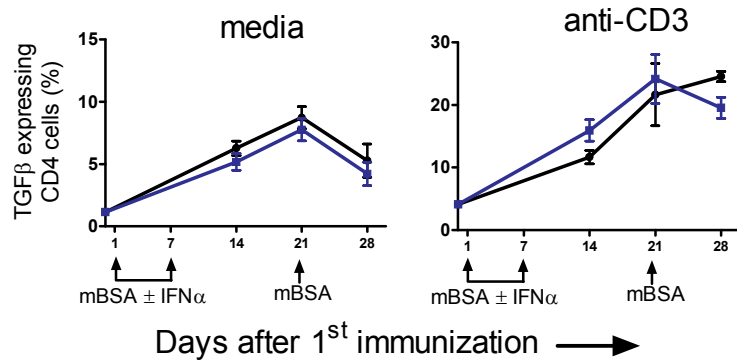

**F**

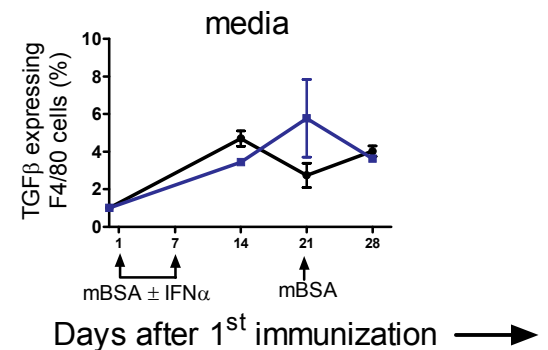

Supplement: Additional file 2: Figure S3 — Effect of IFN-α on intracellular cytokine expression in mock, mBSA and anti-CD3-restimulated T cells and mock and mBSA-stimulated macrophages from splenocytes during AIA. Spleens were collected from mice at days 0, 14, 21, and 28 during the course of AIA and restimulated with complete media (mock) or mBSA (50 μg/ml) or anti-CD3 (1 μg/ml) for 24 hours. Brefeldin A (5 μg/ml) and monensin (1 μg/ml) were added 5 hours before FACS analysis. (A-F) show percentage IFN-γ-, IL-17A-, and TGF-β-expressing spleen cells of gated CD4+(A-C) and F4/80+ cells (D-F). The data shown are mean values ± SEM of pooled data from two independent experiments (n ≥ 5) with similar results (**P < 0.01). Blue line, mice immunized in the presence of IFN-α; black line, control. [file ar4326-S2.pdf]

Figure S2

● PBS treated group (control)

■ IFN $\alpha$  treated group**A**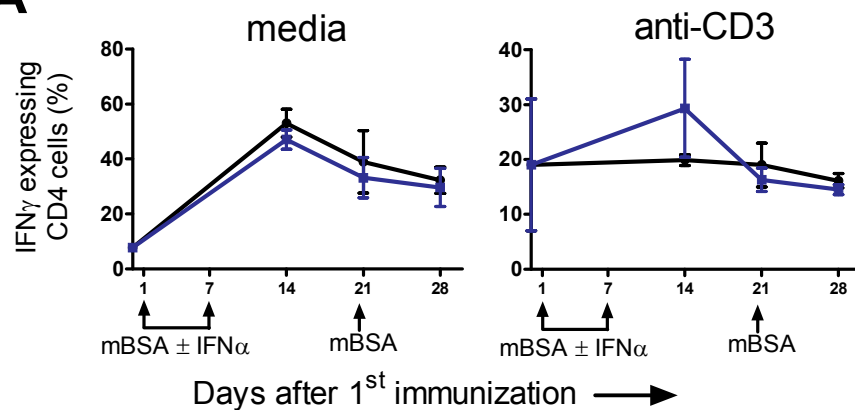**D**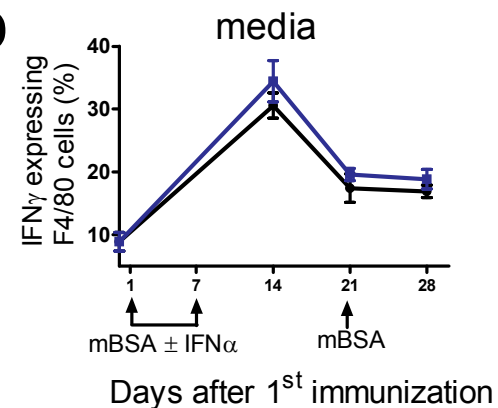**B**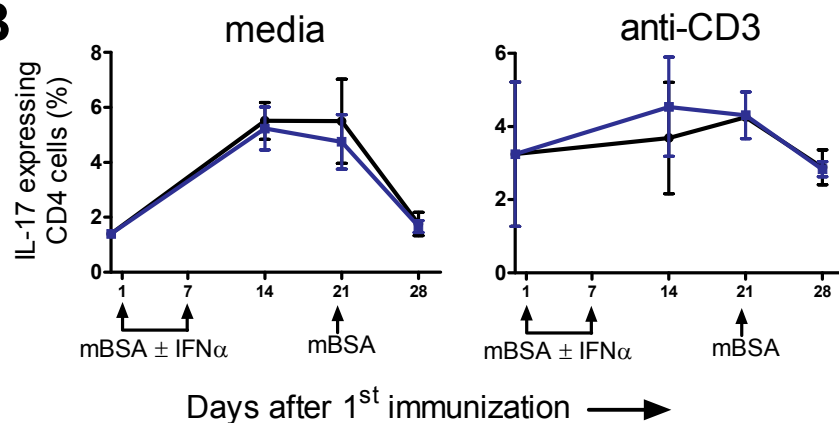**E**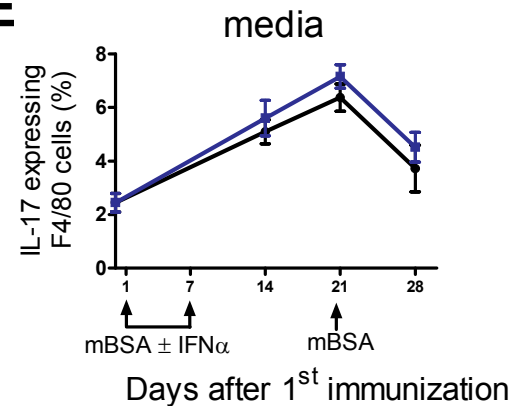**C**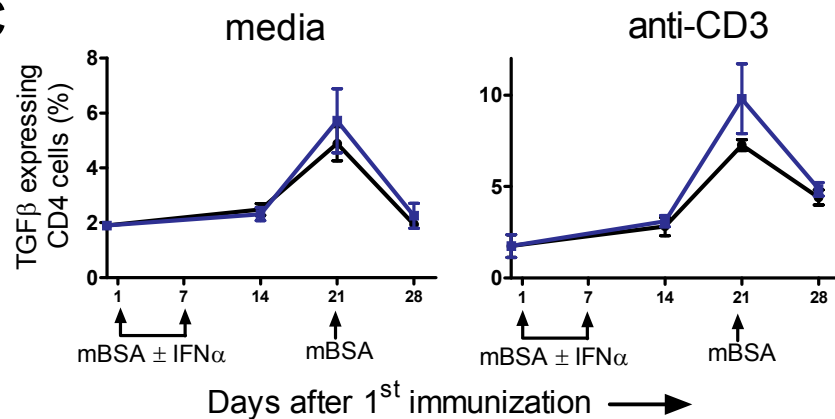**F**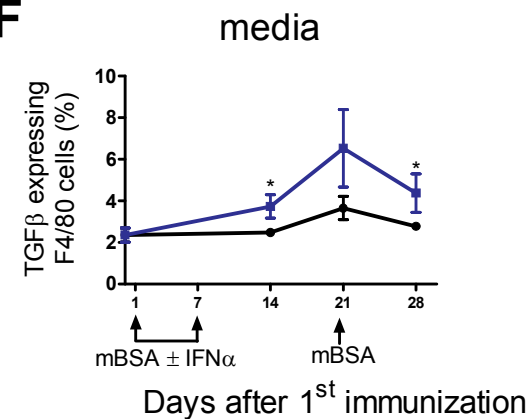

Supplement: Additional file 3: Figure S2 — Effect of IFN-α on intracellular cytokine expression in mock-stimulated macrophages and mock- and anti-CD3-stimulated T cells from lymph nodes during AIA. Draining lymph node cells were collected from mice at days 0, 14, 21, and 28 during the course of AIA and restimulated with complete media (mock) or anti-CD3 (1 μg/ml) for 24 hours. Brefeldin A (5 μg/ml) and monensin (1 μg/ml) were added 5 hours before FACS analysis. (A-F) Percentage IFN-γ-, IL-17A-, and TGF-β-expressing lymph node cells of gated CD4+ (A-C) and F4/80+ cells (D-F). The data shown are mean values ± SEM of pooled data from two independent experiments (n ≥ 5) with similar results (*P < 0.05). Blue line, mice immunized in the presence of IFN-α; black line, control. [file ar4326-S3.pdf]
